# Antimicrobial Effects of Biogenic Nanoparticles

**DOI:** 10.3390/nano8121009

**Published:** 2018-12-05

**Authors:** Priyanka Singh, Abhroop Garg, Santosh Pandit, V. R. S. S. Mokkapati, Ivan Mijakovic

**Affiliations:** 1 The Novo Nordisk Foundation Center for Biosustainability, Technical University of Denmark, 2800 Kgs. Lyngby, Denmark; prisin@biosustain.dtu.dk (P.S.); abhgar@biosustain.dtu.dk (A.G.); 2 Systems and Synthetic Biology Division, Department of Biology and Biological Engineering, Chalmers University of Technology, 41296 Chalmers, Sweden; pandit@chalmers.se (S.P.); ragmok@chalmers.se (V.R.S.S.M.)

**Keywords:** antibiotics, nanoparticles, biogenic nanoparticles, antimicrobial, antibiotic resistance, multidrug resistant (MDR) microorganisms

## Abstract

Infectious diseases pose one of the greatest health challenges in the medical world. Though numerous antimicrobial drugs are commercially available, they often lack effectiveness against recently developed multidrug resistant (MDR) microorganisms. This results in high antibiotic dose administration and a need to develop new antibiotics, which in turn requires time, money, and labor investments. Recently, biogenic metallic nanoparticles have proven their effectiveness against MDR microorganisms, individually and in synergy with the current/conventional antibiotics. Importantly, biogenic nanoparticles are easy to produce, facile, biocompatible, and environmentally friendly in nature. In addition, biogenic nanoparticles are surrounded by capping layers, which provide them with biocompatibility and long-term stability. Moreover, these capping layers provide an active surface for interaction with biological components, facilitated by free active surface functional groups. These groups are available for modification, such as conjugation with antimicrobial drugs, genes, and peptides, in order to enhance their efficacy and delivery. This review summarizes the conventional antibiotic treatments and highlights the benefits of using nanoparticles in combating infectious diseases.

## 1. Introduction

The term ‘antibiotic’ hails from the word ‘antibiosis’ (meaning against life). Antibiotics are chemical compounds, which can either kill or inhibit the growth of microorganisms. Antibiotics can be classified as antibacterial, antifungal, and antiviral, depending on their target group. However, generally speaking, the term antibiotic is most commonly used to describe antibacterial compounds [1]. For decades, antibiotics have been used to treat diseases, as well as for providing support in various medical procedures ranging from organ transplant to chemotherapy. Various classes or generations of antibiotics have been developed depending upon developing MDR and their mode of resistance. The widely known antimicrobial mechanism of antibiotics includes, inhibition of enzymes, interference in DNA, RNA and protein synthesis, and disruption of membrane structure [2]. A world without antibiotics is difficult to imagine. However, this could turn into a reality owing to the emergence of antibiotic resistance in microorganisms [3]. As aptly described by González-Candelas et al., ‘Antibiotic resistance represents one of the best examples of natural selection in action; and also one of the major hurdles in humankind’s fight against infectious diseases’ [4]. The development of drug resistance in microorganisms leads to usage of high drug doses, higher toxicity treatments, longer stays in hospitals, and an increase in mortality [5]. There are various factors which contribute towards antibiotic resistance in microorganisms, such as misuse and overuse of antibiotics, their extensive agricultural use, and availability of fewer new antibiotics [6]. Furthermore, the ease of transportation (of affected individuals and food commodities) in today’s era helps spreading of pathogenic microorganisms farther and faster around the globe [7].

Apart from the negative social and economic effects on society, antibiotic resistance poses a serious threat of spread of epidemic infections [7]. The World Health Organization (WHO) has declared antimicrobial resistance (AMR) as one of the ‘biggest threats to global health’ [8]. Around 25,000 deaths per annum have been estimated in the European Union because of AMR [3]. Globally, the estimated number of deaths due to multidrug resistant microorganisms is around 700,000 per year [9]. The possible known mechanisms of antibiotic resistance in bacteria are; (1) reduced uptake of antimicrobial drugs and/or increased efflux of drugs, (2) alterations of antibiotic target, (3) development of drug degrading/modifying enzymes in microorganisms, and (4) formation of biofilm layer which surrounds the bacteria and avoids its exposure to antibiotics [10]. These possibilities ultimately result in either less accumulation of drugs in microbial cells or short intracellular residence of drugs, due to which the therapeutic levels of drugs cannot be easily achieved [11]. Consequently, a higher amount and repeated administration of drugs is required, leading to adverse side effects on human beings and animals.

Pathogenic microorganisms have developed resistance against almost all the types of antibiotics currently being used [12]. Most importantly, there have been no reports on the development of any new antibiotics class in the past few decades. In addition, antibiotics innovation and commercialization is an expensive and long process, which includes discovery of new antibiotics, several clinical trials, and licensing [13]. This situation is compounded by the fact that bacterial resistance can emerge quickly to any new antibiotics, resulting in a reduction in antibiotic use and a decline in sales. Thus, lack of antibiotics development will ultimately result in an increased risk of death from infections following surgeries such as organ transplants or chemotherapy [14]. Therefore, there is an imperative need to develop new drugs to tackle these problems.

To answer these problems, scientists became interested in achieving a rapid diagnostic and targeted therapy by either completely avoiding or modifying the use of conventional antibiotics. This search led to investigating the metals such as silver, copper, zinc, and titanium, which are originally antimicrobial in nature. Metals have been used as antimicrobial agents from centuries. Unlike antibiotics, metals act against microorganisms through several different mechanisms such as membrane disintegration, damage of cellular components (DNA, protein and electron transport chain), and reactive oxygen species (ROS) generation [15]. The emergence of nanotechnology helped in understanding and exploring the unique properties of these metals. Conversion of bulk metal-to-metal nanoparticles demonstrated the enhancement of all the properties of parent metal at nano scale [16]. Transformation of bulk element to nano level, not only reduces its size, but also leads to the formation of different shapes at nano level, such as spherical, triangular, truncated triangle, octahedral, rod, and flower-shaped [17,18]. This variation in geometry facilitates applications in various fields. Especially, it is very advantageous for antimicrobial applications, since the antimicrobial action of nanoparticles is directly proportional to the surface area available for interaction with biological components. Thus, the metallic nanoparticles became one of the most promising choices to overcome the microbial resistance and fight MDR microorganisms [11].

**Figure 1:**
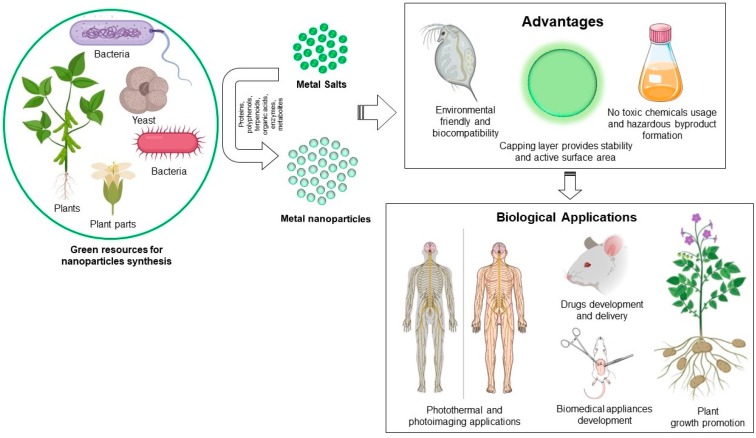
Green synthesis of metallic nanoparticle, their advantages and biological applications.

To produce the metallic nanoparticles, several conventional methods have been in use for decades. For instance, physical methods such as melt mixing, laser ablation, physical vapor deposition, sputtering, and chemical methods like thermolysis, photoreduction, microemulsion and sol-gel. These methodologies often result in instability of nanoparticles, attachment of toxic substances on nanoparticle surface, and production of hazardous byproducts. For instance, to produce silver nanoparticles (AgNPs) chemically, a reducing agent (borohydrite), capping agent (starch, polyethyl glycol), and other stabilizing agents are required. By contrast, “green” methodologies have overcome all these limitations [19]. Green methodologies involve biogenic synthesis of metallic nanoparticles by using biological resources such as microorganisms and plants [20,21,22]. Microorganisms usually exhibit a process called bioreduction, which involves the accumulation of metallic ions in order to reduce their toxicity. Microorganisms bioreduce intracellularly with the help of various reducing species present either inside the cell and on the cell wall, or extracellularly by different metabolites. Plants also possess the reducing capability because of various flavonoids, proteins, and water-soluble biomolecules. The advantages of green synthesis include: (1) production of stable nanoparticles, (2) biocompatible coating on the nanoparticles’ surface which provides additional active surface area for interaction in the biological environment, (3) no hazardous byproduct formation, (4) additional reduction or stabilizing agents are not required, which ultimately makes the process economical (Figure 1) [23,24]. The stability and biocompatibility of green nanoparticles corresponds to their capping layer, which usually form during synthesis of biogenic nanoparticles, and originates from the corresponding biological extracts used for synthesis. This layer affects the biological activity of nanoparticles and is useful in long-term stability. Huang et al. demonstrated that nanoparticles formed from microorganisms through nucleation and surface growth could be entrapped by the additional surface (capping layer), often exhibiting excellent stability [25]. Despite the fact that biogenic metallic nanoparticles are biocompatible in nature with high stability and amenable for biomedical applications, a balance between price, process, and scalability is still a considerable challenge. Especially for microorganisms involved in biogenic nanoparticles production, there is a requirement of sophisticated instruments throughout the process for the maintenance, production, and purification of nanoparticles. For instance, freezers are required for microorganisms’ preservation, incubators with temperature and shaking control are required for nanoparticle production, and centrifuges are required for purification of nanoparticles. All these heavy instruments required for the complete process of nanoparticle production and purification make the methodology comparatively expensive [26,27]. In the case of plants, the requirement of natural resource management, which includes plant culturing and maintenance, is an important issue that needs to be addressed [28,29]. However, the advantages of biogenic metallic nanoparticles over physiochemically-obtained nanoparticles cannot be over looked for future research and commercialization in the field of antimicrobial applications.

In this review, we focused on conventional antibiotics, developing drug resistance, nanoparticle development, and overcoming drug resistance problems. We also focused on the biogenicity of metallic nanoparticles and their future perspectives.

## 2. Microbial Resistance to Antimicrobial Drugs 

### 2.1. Conventional Antibiotics

Penicillin was the first antibiotic to be discovered in 1928, which marked the beginning of the modern era of antibiotics [6]. Antibiotics can be classified on the basis of their mode of action, spectrum of action, or their chemical structure. For example, antibiotics can either be bactericidal (lethal to bacteria) or bacteriostatic (causing growth inhibition of bacteria). The broad-spectrum antibiotics target both the Gram-negative and Gram-positive bacteria, while the narrow spectrum antibiotics target only one of them [30]. Based on their molecular structures, antibiotics can be classified as β-lactams, macrolides, tetracyclines, quinolones, aminoglycosides, sulphonamides, glycopeptides, and oxazolidinones [1].

The β-lactam antibiotics interfere with the cell wall synthesis in bacteria by binding to penicillin binding protein (PBP). The function of PBPs is to cross-link the peptide units in the peptidoglycan layer. Binding of β-lactams to PBPs leads to the inhibition of the latter, and subsequently cell lysis. The β-lactam antibiotics are further divided into penicillins, cephalosporins, monobactams, and carbapenems. In the late 1960s, the emergence of penicillin-resistant bacteria was observed. These bacteria were able to synthesize β-lactamases, enzymes that could degrade β-lactam antibiotics. The discovery of carbapenems circumvented this problem, as this new class of β-lactams was insensitive to the β-lactamases. Amongst all the known β-lactams, carbapenems exhibit the broadest spectrum of activity [1]. Unfortunately, the emergence of carbapenem resistance was also reported in bacteria [31]. Glycopeptides also target the bacterial cell wall synthesis, but in addition to blocking the PBPs, they also inhibit peptidoglycan synthesis [1]. A detailed account of recent developments in glycopeptide antibiotics has been published elsewhere [32]. The macrolides, tetracyclines, aminoglycosides, and oxazolidinones inhibit the bacterial growth by targeting protein synthesis in the cells. Macrolides bind to the 50S ribosomal subunit and inhibit the elongation of mRNA during translation, thus halting protein synthesis [1]. Oxazolidinones also bind to the 50S ribosomal subunit, but unlike the macrolides, inhibit protein synthesis by impeding the formation of 70S translation initiation complex [33]. Together, these two classes form the 50S inhibitors group. Tetracyclines and aminoglycosides, the 30S inhibitors group, bind to the 30S ribosomal subunit denying aminoacyl-tRNAs access to the ribosome and subsequently inhibiting protein synthesis. While macrolides and tetracyclines are typically bacteriostatic, aminoglycosides are broadly bactericidal in their mode of action [1]. Nucleic acid (DNA and RNA) synthesis is fundamental to a cell’s survival. Quinolones inhibit bacterial growth by blocking the action of DNA helicases, which are indispensable for unwinding the double helical structure of DNA prior to DNA replication or repair. Additionally, quinolones also interfere with the functions of topoisomerase II and topoisomerase IV in bacteria leading to a negative effect on RNA polymerase, thereby inhibiting RNA synthesis [1]. Sulfonamides structurally mimic para-aminobenzoic acid (PABA), a substrate for the synthesis of folic acid in bacterial cells. Folic acid is indispensable for nucleic acid (DNA) synthesis, thus by competing with PABA and blocking folic acid synthesis; sulfonamides inhibit cell division and cause growth inhibition in bacteria. Unfortunately, resistance to these conventional antibiotics has been reported in bacteria, making it difficult to treat the infections caused by these bacteria [34].

### 2.2. Developing Resistance to Antimicrobials

Recently-developed multidrug resistant (MDR) microorganisms includes: vancomycin resistant *Staphylococcus aureus* and *Enterococcus* sp. such as *E. faecalis* and *E. faecium* [35], penicillin resistant *Streptococcus pneumonia*, multidrug resistant *Mycobacterium tuberculosis*, *Salmonella enterica*, *Pseudomonas aeruginosa*, *Vibrio cholera*, *Acinetobacter baumannii*, and carbapenem resistant *Enterobacteriaceae* [9]. Broadly speaking, bacteria develop drug resistance by acquiring the drug resistance genes, which is followed by the expression of these resistance genes, and selection of the cells expressing the resistance genes. The acquisition of resistance genes can occur via horizontal gene transfer (HGT) by transduction, transformation, or conjugation [36]. Another possibility of acquiring the resistance genes is by spontaneous mutation in the existing genes [37]. When a microbe, which already has a drug resistance gene, acquires another type of drug resistance gene, such microbes then become multi drug resistant (MDR). Next, the acquired resistance genes are expressed when the microbes possessing them are exposed to antimicrobial drugs. Finally, a selection pressure for microbes expressing a resistance gene leads to a widespread resistance towards that antimicrobial. This could happen when the microbes are not eliminated completely upon exposure to the drug, resulting in a positive selection pressure for the drug resistant microbes. For example, a positive selection pressure for microbes expressing resistance genes occurs when a patient misses a scheduled dose of the antimicrobial or takes an insufficient number of doses (poor patient compliance). Consequently, the microbes get exposed to the drug but are not completely eliminated. Poor patient compliance plays an even more significant role in developing drug resistance against drugs with short elimination half-lives. Because the time required for removal of these drugs from the host body is short, it is necessary to replenish the drug in short intervals accompanied by a higher number of doses for complete eradication of the microbe [12]. 

However, administration of an appropriate number of doses at appropriate intervals does not eliminate the positive selection pressure for drug resistance. The clinical outcomes of time-dependent antibiotics are measured as a function of t > MIC (minimum inhibitory concentration), which is defined as the time duration, between the doses, for which the drug concentration in plasma is more than its MIC. Thus, persistent plasma concentration of a time-dependent antibiotic between zero and its MIC for a long time can lead to the development of resistance against the drug. This especially concerns antibiotics with long elimination half-lives such as β-lactams, tetracyclines, and clindamycin [5]. The clinical outcomes of concentration dependent antibiotics are measured as a function of C_max_/MIC, which is defined as the ratio of maximum drug concentration in plasma to its MIC, per dosing interval. Thus, a drop in C_max_/MIC value below a target threshold during a dosing interval can lead to the development of resistance against the drug, independent of its elimination half-life. Vancomycin, aminoglycosides, and quinolones are some examples of the concentration-dependent antibiotics [5].

### 2.3. Mechanisms of Drug Resistance to Antimicrobials

#### 2.3.1. Decreased Uptake and Efflux Pumps

Decreased uptake and increased efflux of a drug does not allow for accumulation of the drug inside the cell to a concentration that is lethal to cells. For this purpose, various bacteria possess resistance genes for specific types of antibiotics. For example, both Gram-positive and Gram-negative bacteria possess the genes for tetracycline efflux pumps TetA, TetB, and TetK. The *tetA* gene is not expressed under native conditions owing to its repression by the repressor protein TetR. Tetracycline binds to TetR, thus inactivating it, which in turn leads to the expression of the *tetA* gene. The TetA efflux pump then flushes out tetracycline, thereby conferring resistance to the bacteria against tetracycline. Other examples of resistance due to increased efflux include resistance against fluoroquinolones in Gram-negative bacteria and resistance against macrolide in Gram-positive bacteria [5]. Examples of decreased uptake of antibiotics include aminoglycoside resistance in Gram-negative bacteria. One of the known vancomycin resistance mechanisms is a thickening of the cell wall [5].

#### 2.3.2. Alteration of Antimicrobial Target

Bacteria can also develop resistance by expressing genes that code for an alternate version of the antibiotic target. These altered substrates usually have lower binding affinity to the antibiotic as compared to the wild type versions, thus decreasing the activity of the antibiotic. For example, resistance against β-lactams in methicillin resistant *Staphylococcus aureus* (MRSA) conferred by *mecA*, which codes for an altered PBP known as PBP2A. The β-lactams have lower binding affinities towards PBP2A than PBP, and therefore, *mecA* confers resistance against all the β-lactams [5,38,39]. Another example is resistance against glycopeptides conferred by the resistance gene *vanA* expressing the enzyme d-alanine-d-lactate ligase. This enzyme modifies the terminal d-ala-d-ala domain of peptidoglycan precursor (target of vancomycin) to d-ala-d-lactate. The affinity of vancomycin towards this modified precursor is about 1000 times lower than the wild type version, thus making the cells expressing *vanA* resistant towards vancomycin [5,40]. Other examples that use this mechanism to develop drug resistance include resistance to sulfonamides in *Escherichia coli*, *Streptococcus pneumoniae*, *Neisseria meningitidis*, resistance to quinolones in Gram-positive and Gram-negative bacteria, and resistance to macrolides, aminoglycosides, and tetracyclines [5,41].

#### 2.3.3. Modification of Antimicrobial Drugs

Bacteria have also been observed to express resistance genes coding for antibiotic modifying enzymes. For example, ACT *N*-acetyltransferase which catalyzes the acetylation of an NH_2_ group of aminoglycoside, the APH O-phosphotransferase which catalyzes the phosphorylation of an OH group of aminoglycoside, and ANT O-adenyltransferase which catalyzes the adenylation of an OH group of aminoglycoside. In all these cases, modification of the antibiotic leads to its decreased binding affinity towards its target, the 30S ribosomal subunit, consequently reducing its antimicrobial activity. Modification and inactivation of chloramphenicol by acetyltransferases is the most common mechanism of developing chloramphenicol resistance. Other antibiotics for which such mechanisms of developing drug resistance are observed include β-lactams, tetracyclines, macrolides, quinolones, and streptogramins [5,42]. 

#### 2.3.4. Production of Competitive Inhibitor

Antibiotic resistance is also acquired by producing a competitive inhibitor of the drug. For example, *S. aureus* and *N. meningitides* produce an increased amount of PABA that competes with sulfonamide for its target, dihydropteroate synthetase, and thus conferring resistance against sulfonamide drugs [5].

#### 2.3.5. Persister Cells

When a small fraction in bacterial population randomly stops or slows down their metabolic activity by expressing the toxin-antitoxin (TA) genes, they become more tolerant to the antimicrobial drug. These cells are known as persisters. Upon exposure to antibiotics, most of the bacterial population is wiped out, leaving behind the persisters. These persistors can cause recurrence of the infection when they resume their metabolic activity [5,36].

#### 2.3.6. Biofilm Formation

Biofilms are formed when bacterial cells immobilize themselves by attaching to a surface such as human tissues and medical implants. It is very difficult to treat the infections associated with biofilms because of the extracellular polymeric substance (EPS) matrix present around the bacterial cells. The EPS matrix is extremely tolerant towards various antibiotics, thus leading to chronic infections in humans [5,43]. The EPS matrix forms a barrier between antibiotics and bacterial cells. The EPS matrix acts a sieve and molecules above a certain size, including antibiotics, cannot pass through it. The antibiotics also get trapped in the EPS matrix because of its negative charge. Furthermore, the EPS matrix contains enzymes that can modify antibiotics and rip them off their antimicrobial activity. It has also been suggested that by reducing the antibiotic concentration below their MIC (and above 0), the EPS matrix could help in development of antibiotic resistance in the bacterial cells [5,44]. Although some antibiotics, such as rifampicin and vancomycin, have been shown to penetrate the EPS matrix, they could not eradicate the slow growing bacterial cells, especially the persister cells [43].

#### 2.3.7. Swarming

Swarming is a type of multicellularity observed in many bacterial species. It happens when groups of highly differentiated cells (swarm cells) come together as a single unit on semisolid surfaces. The planktonic cells become elongated and develop multiple flagella. These swarm cells remain in each other’s vicinity and migrate together, like a raft. The swarm cells have been shown to be highly resistant to multiple antibiotics. However, sub culturing the swarm cells in liquid medium causes them to revert back to planktonic cells, as well as restoring their antibiotic susceptibility [5,38].

#### 2.3.8. Intracellular Microbes

Being inside the host cell, the intracellular microbes are shielded from the antimicrobial drugs because of the limited capacity of the drugs to enter the host cell [5].

In recently published reviews, a more detailed account on the mode of action of antibiotics and different mechanisms of resistance against antibiotics is available [34,45].

## 3. Promising Biogenic Metallic Nanoparticles for Antibacterial Applications

**Figure 2:**
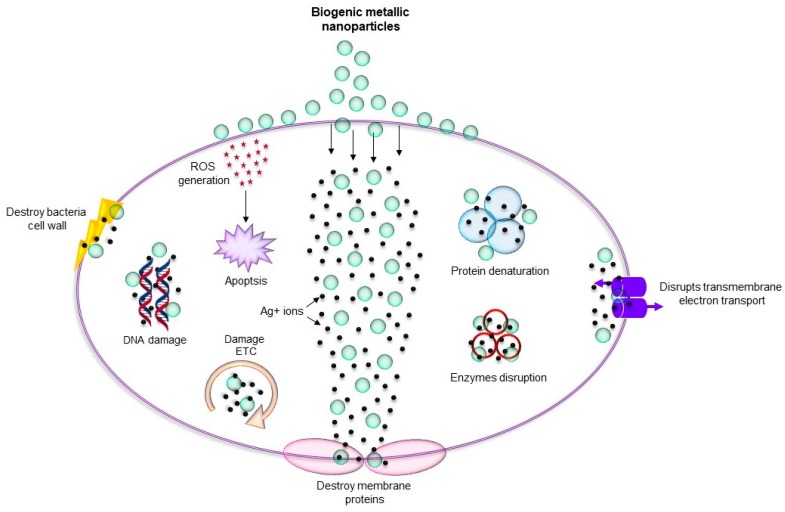
Various mechanism of antimicrobial activity of biogenic metallic nanoparticles. ROS: reactive oxygen species.

As discussed above, metallic nanoparticles due to their shape-and-size-dependent tunable properties became central focus for many biomedical applications including antimicrobial. Metallic nanoparticles such as silver, copper, titanium, zinc, and iron can be used against MDR microorganisms due to their antimicrobial nature [11,46]. Importantly, biogenic nanoparticles are mainly utilized for antimicrobial applications due to their long-term stability and biocompatibility. The mechanisms behind the antimicrobial effect of these nanoparticles are oxidative stress, metal ion release, and non-oxidative stress occurring simultaneously (Figure 2) [47]. There are several examples where green metallic nanoparticles obtained from microorganisms have been explored for antimicrobial applications against many pathogenic microorganisms. For instance, biogenic AgNPs obtained from *Brevibacterium frigoritolerans* DC2 [48], *Sporosarcina koreensis* DC4 [49], and *Bhargavaea indica* DC1 [18], showed antimicrobial activity against *Vibrio parahaemolyticus*, *Salmonella enterica*, *Bacillus anthracis*, *Bacillus cereus*, *Escherichia coli*, and *Candida albicans*. Copper nanoparticles (CuNPs) obtained from *Sida acuta* showed antimicrobial activity against *Escherichia coli*, *Proteus vulgaris*, and *Staphylococcus aureus* [50]. In addition, these nanoparticles showed enhancement in the antimicrobial efficacy of conventional antibiotics such as lincomycin, oleandomycin, vancomycin, novobiocin, penicillin G, and rifampicin, when applied together. Research on zinc oxide also revealed its antibacterial activity against *S. aureus*, *E. coli*, and *P. aeruginosa* [51]. Thus, the findings suggest that combining the current antibiotics with green metallic nanoparticles can be further helpful for enhancing their antimicrobial activity. Moreover, a comparative study between biological and chemical nanoparticles demonstrated that the biological nanoparticles exert higher antimicrobial effect than the chemically synthesized nanoparticles. For example, Sudhasree et al. proposed that the biological synthesized nickel nanoparticles from *Desmodium gangeticum* are more monodispersed and have higher antioxidant, antibacterial, and biocompatible activities in LLC PK1 (epithelial cell lines) than chemically synthesized nanoparticles. Specifically, in terms of antibacterial activity, they tested both the nanoparticles against *S. aureus*, *K. pneumonia, P. aeruginosa*, *V. cholerae*, and *Proteus vulgaris*, and found that chemically synthesized nickel nanoparticles were not at all active against *K. pneumonia*, *P. aeruginosa* and *P. vulgaris*, whereas biological nanoparticles showed antimicrobial activity against these microorganisms. For *S. aureus*, chemical nanoparticles were less active than the biological ones. However, in the case of *V. cholerae*, chemical nanoparticles were more effective [52]. Mohammed et al. also described how biologically synthesized zinc nanoparticles have more antimicrobial potential against *Salmonella typhimurium* ATCC 14028, *B. subtilis* ATCC 6633, and *Micrococcus luteus* ATCC 9341 compared with chemically synthesized zinc nanoparticles [23]. Table 1 provides an overview of several types of biogenic nanoparticles, their source, and any reported antimicrobial activity. 

However, this list is not exhaustive, and only a few seminal studies are mentioned here. From past few decades, nanoparticles, especially coating of nanosilver, are being used in bone prostheses, dental implants and surgical instruments as an antibacterial preventive measure and as coating on wound dressing to combat the microorganisms in wounds [53,54]. These nanoparticles target the bacterial cells and disturb the crucial function of cell membrane such as membrane respiration and membrane permeability [55,56]. Furthermore, they react with intracellular components such as proteins and nucleic acids, and inhibit cell division and gene transfer [55,56]. There are many reports showing the antimicrobial activity of various nanoparticles, mainly silver, zinc, copper, titanium, magnesium, and gold [57,58]. The mechanism of action of nanoparticles and antibiotics seems to be similar in the case of interference in the synthesis of DNA, RNA, and protein, as well as membrane disruption [2,53,55]. However, most of these metallic nanoparticles exhibit antimicrobial activity through multiple mechanisms, which decrease the possibility of development of resistance against them in microorganisms [56]. To develop resistance towards such nanoparticles, microbial cells would need to acquire multiple simultaneous gene mutations, which is not very probable. Furthermore, synthesizing such nanoparticles by using the green way would result into proteins, polysaccharides, and small bioactive compounds binding to the nanoparticles, which further enhance their antimicrobial activity towards the MDR microorganisms. In this section, we discuss a few metallic nanoparticles that are synthesized by green method(s) and their effect on different pathogenic microorganisms.

### 3.1. Gold Nanoparticles (AuNPs)

One of the most widely studied biogenic nanoparticles are gold nanoparticles (AuNPs). Predominantly, the shape of the AuNPs is spherical [59], triangular [60], and hexagonal [61], though rod-shaped nanoparticles were also reported in various studies. AuNPs are synthesized either from the whole plant or by the combination of various components that act as reducing agents. Interestingly, the type of extracts that are used as bioreductants defines the size and shape of synthesized nanoparticles. AuNPs synthesized from *Galaxaura elongate* is one important example where a wide range of size (4–77 nm) and shapes (spherical, rod, triangular, hexagonal) of nanoparticles were obtained [62]. Another important discovery is the effect of pH on the size of AuNPs. It was reported that nanoparticles with core size 6 nm and 18 nm were obtained at pH 9 and pH 2 respectively, from mango peel extract [63]. AuNPs are known for their biocompatibility to microbial cells with no bacteriostatic or bactericidal activity. However, antibiotics integrated AuNPs are shown to have strong bactericidal effect against the drug resistant bacteria. The ampicillin bound AuNPs has been shown to damage ampicillin resistant bacteria, including MRSA, *P. aeruginosa*, *Enterobacter aerogenes*, and *E. coli* K-12 sub-strain DH5-alpha [64] by multiple mechanisms. AuNPs-AMP can overwhelm the high concentrations of beta-lactamase expressed by these bacteria and in addition, AuNP-AMP inhibits the transmembrane pump that catalyzes drug efflux from the bacterial cell [64].

### 3.2. Silver Nanoparticles (AgNPs)

AgNPs have remarkable bactericidal and fungicidal properties, that have been exploited in pharmaceutical industry, paints, ointments, food, fabrics, and packaging industries [65]. Large-scale green synthesis of different shapes and sizes of AgNPs from plants, bacteria, fungi, and yeast has been studied extensively [23]. The basic antibacterial mechanism of AgNPs has been shown to be either due to the release of silver ions or due to the intracellular deposition of nanoparticles [58,66]. The detailed mechanism mainly involves cell membrane damage, disruption of energy metabolism, generation of oxidative stress due to ROS formation, and inhibition of transcription. Silver ions released from AgNPs have been shown to interact with sulfur- and phosphorus-containing groups of proteins in the cell wall and plasma membrane of bacteria [67]. The initial interaction of silver ions with microbial cells starts with the binding of cationic silver with the negatively charged microbial cell, which leads to the formation of multiple pores in the cell membrane and outflow of the intracellular contents. This also causes an electrochemical imbalance in the cells and allows the silver ions to pass through the plasma membrane into the cytoplasm of the bacterial cell and interact with the intracellular components resulting in permanent cell damage [55]. Silver ions also have been shown to inhibit the activity of proteins and enzymes that are essential for ATP production, inhibit respiratory enzymes leading to the production of ROS, damage RNA and DNA, and destabilize and disrupt the outer membrane. Nanoparticles, owing to their small size with large surface area, have a high possibility to cross the peptidoglycan and cell membrane [10,68]. This phenomenon has been described as a rationale for the higher sensitivity of the Gram-negative bacteria towards nanoparticles, as compared to the Gram-positive bacteria having a thicker peptidoglycan layer [19]. The thickness and crosslinking of peptidoglycan in the Gram-positive bacterial cell wall provide more resistance against the penetration of nanoparticles. Many reports with antibacterial activity of AgNPs have correlated their toxicity with size and shape of the particles [69]. The nanoparticles with more surface area have been shown to release silver ions at a higher rate, which is an important factor for high antibacterial activity [70]. Antibacterial activity of AgNPs have been studied against the multidrug resistant bacteria such as *P. aeruginosa*, *E. coli*, *Streptococcus pyogenes*, *S. aureus*, *Klebsiella pneumoniae*, *Salmonella* species, and *Enterococcus* species [71,72]. This bactericidal effect, mostly, is attributed to the inhibition of cell wall synthesis, protein synthesis mediated by the 30S ribosomal subunit, and nucleic acid synthesis. Furthermore, AgNPs have also been shown to enhance the antimicrobial activity of antibiotics such as penicillin G, amoxicillin, vancomycin, clindamycin, and especially erythromycin, against *S. aureus* and *E. coli* [73]. In addition to that, silver carbene complexes encapsulated in nanoparticles have been shown to be effective against multidrug resistant bacteria, including MRSA, multidrug resistant *A. baumannii* (MRAB), *P. aeruginosa*, *Burkholderia cepacia*, and *K. pneumoniae* [74]. The strong bactericidal effect of AgNPs against the multidrug resistant bacteria is mostly due to their multiple mechanisms to disrupt microbial cells. Despite having multiple mechanisms for antibacterial effects, a recent study involving a pretreatment of bacterial cells with sublethal concentration of AgNPs showed lesser membrane damage, lowered levels of intracellular ROS and higher amount of intracellular ATP when bacterial cells were further exposed to ampicillin. This suggests that the pretreatment of bacterial cells with sub-lethal concentrations of AgNPs leads to long-lasting responses that enhance the antibiotic stress resistance in bacteria at multiple levels [75].

### 3.3. Zinc Oxide Nanoparticles (ZnO-NPs)

Zinc oxide nanoparticles (ZnO-NPs) are synthesized using different biological resources as reducing agents [76]. They are nontoxic, semiconducting material with good photocatalysis and high transparency. ZnO-NPs are synthesized from different parts of plants such as leaves, roots, rhizomes, fruits, flowers, and bark [77]. ZnONPs show a potential antibacterial activity [78] and good photo degradation and have applications in drug delivery [79] and anticancer therapy [80]. ZnO-NPs are also widely tested metallic nanoparticles for their antimicrobial purpose. The wide range of both Gram-positive and Gram-negative bacteria such as *E. coli*, *Listeria monocytogenes*, *Salmonella*, and *S. aureus* have demonstrated sensitivity towards ZnO-NPs [81,82]. ZnO-NPs treatment of bacterial cells leads to ROS generation, lipid peroxidation, membrane leakage of reducing sugars, proteins, DNA, and cell viability [83]. ZnO-NPs has been shown to produce ROS such as super oxide anion and hydrogen peroxide in cells [84,85]. ROS causes membrane leakage of proteins and nucleic acids by enhancing lipid peroxidation on membrane. Additionally, Zn^+2^ ions released from the nanoparticles also damage the cell membrane and interact with intracellular components [86,87]. Recently, ZnO-NPs were shown to inhibit the growth of carbapenem-resistant *A. baumannii* by producing ROS and causing membrane damage, suggesting that ZnO-NPs might be developed as an alternative to carbapenems (beta-lactam) [83].

### 3.4. Copper Nanoparticles (CuO-NPs)

Cupric oxide nanoparticles (CuO-NPs) gained critical importance due to their applications in anti-microbial activity, pharmaceutical industry, cosmetics, transport, power, and farming [88]. It is relatively easy to produce CuO-NPs by chemical means, but with many disadvantages like low potency, high toxicity, environmentally unfriendliness, and high expense. CuO-NPs are synthesized from various biogenic means like polysaccharides such as pectin, chitosan, alginate, leaf extracts, bacteria and so on. Unlike gold, silver, and other nanoparticles, it has been a challenge to produce stable CuO-NPs due to their proneness to oxidation when exposed to an aqueous medium [89]. Though there are a few reports on CuO-NPs production under inert conditions [90] from copper salts, there are very limited reports that suggest the synthesis of metallic CuO-NPs in noninert conditions. Compared to other nanoparticles, the biogenic synthesis of CuO-NPs is relatively new and ways are being explored to make it with ease and ecofriendly. The mechanism behind the antibacterial activity of CuO-NPs is believed that electrostatic attraction between Cu^+2^ and plasma membrane helps in damaging the membrane and killing cells [91,92]. The Cu^+2^ ions are energetically easier to move across a lipid bilayer and upon being taken up by the cell, lead to ROS production, lipid peroxidation, and protein oxidation [92]. CuO-NPs were shown to have strong antimicrobial activity against both Gram-positive and Gram-negative bacteria [92,93]. The broad-spectrum antimicrobial efficacy of CuO-NPs suggested the possible use in wound healing treatment, such as in bactericidal plasters and bandages, due to its strong bactericidal effect and illegible sensibility of human tissues to copper compounds [94,95].

### 3.5. Titanium Dioxide Nanoparticles (TiO_2_-NPs)

Titanium dioxide nanoparticles (TiO_2_-NPs) possess interesting optical, dielectric, antibacterial, and catalytic properties that makes them interesting for their usage in various catalyst industry [96], sensors [97], biosensors [98], solar cells [99], and as image-contrast agents in medical diagnostics [100]. TiO_2_-NPs with different morphologies like nanorods and nanotubes are commonly synthesized using different reducing and stabilizing agents [101]. Hydrothermal processing is another approach due to its cost effectiveness and simplicity [102], nevertheless, green routes need to be developed to have a reliable supply in sufficient quantities without any harmful effects on the environment. TiO_2_-NPs are synthesized from plants [103], fungus [104], and piper betel leaf [105]. TiO_2_-NPs also exhibit antimicrobial activity by multiple mechanisms suggesting that the possibility of development of resistance by microbial cells against these nanoparticles is very low [10]. TiO_2_-NPs have been well demonstrated to have bactericidal effect against *E. coli*, *P. aeruginosa*, *S. aureus*, and *E. faecium* [106,107]. One of the mechanisms by which TiO_2_-NPs kills microorganisms is by generating ROS with the exposure of near to ultra-violet radiation [107]. The generated ROS disrupt the cell membrane interfering with the oxidative phosphorylation, which leads to cell death. A recent report suggested that exposing cells to TiO_2_ photocatalysis rapidly inactivates the regulatory signaling level, efficiently decreases the coenzyme-independent respiratory chains, lowers ability to take up and transport iron and phosphorous, and lowers the capacity for the biosynthesis and degradation of heme (Fe-S cluster) groups [11,106]. 

### 3.6. Magnesium Oxide Nanoparticles

Like other nanoparticles, magnesium oxide nanoparticles (MgO-NPs) also generates the ROS and is the major mechanism behind its antimicrobial activity [70]. Like other nanoparticles, MgO-NPs physically interact with the cell’s surface and disrupt the membrane integrity leading to membrane leakage [10]. In addition, they damage the cells by irreversible oxidation of intracellular biomolecules. However, another study demonstrated that MgO-NPs exhibit excellent antibacterial activity in the absence of ROS and lipid peroxidation. The authors suggested that antibacterial activity of MgO-NPs is correlated with the interaction of nanoparticles with the microbial cell membrane, pH change, and release of Mg^+2^ [108]. Furthermore, unlike other nanoparticles, the antimicrobial activity of MgO-NPs has been demonstrated to be due to adsorbing halogen molecules onto the surface of the MgO [10]. 

## 4. Concluding Remarks and Future Perspectives

In summary, we would like to conclude that due to poor diagnostics and overdose and incapability of drugs, microorganisms are commonly able to develop resistance against antibiotics. The infections caused by MDR microorganisms are a serious global healthcare issue. To address these problems, biogenic metallic nanoparticles were developed and had proven strong efficacy against various MDR pathogens, either individually or in combination with antibiotics. However, in order to use these nanoparticles for therapeutic applications, some important facts that need to be considered are nanoparticles distribution, their bioavailability, active targeting, and nanoparticles excretion from the body if taken as drug carrier for treating site-specific infections [109]. 

Owing to the antimicrobial nature of metallic nanoparticles, the applications are not only limited to the biomedical area, but can also be extended to water treatment, textiles, food packaging, cosmetics, agriculture (nanopesticides and nanofertilizers), self-cleaning coatings on mobiles phones, washing machines, and computer keyboards. However, the biogenic nanoparticles have not yet been commercialized for these applications. The true challenge for biogenic nanoparticles is finding the right balance between the production cost, scalability, and their applicability. Hence, in this respect, a great deal of research will be required to focus on economical ways of biogenic nanoparticles development which will make them easily available for all kinds of future applications relevant to either antimicrobial era or other.

## Figures and Tables

**Table 1:** Overview of several types of biogenic nanoparticles, their source and reported antimicrobial activity.

Origin Plant	NPs Type	Shape of NPs	Size Range of NPs (nm)	Anti-Microbial Effect	References
*Phyllanthus amarus*	CuO	Spherical	20	Anti-microbial against *B. subtilis*	[110]
*Geranium leaves*	Ag	Quasilinear	40	Antimicrobial	[111]
*Avena sativa*	Au	Rod-shaped	5–20	No data available	[112]
*Catharanthus roseus*	TiO_2_	No typical shape	25–110	No data available against bacteria	[113]
*Camellia Sinensis*	ZnO	Triangular/spherical	30–40	Anti-bacterial	[114]
**Bacteria**					
*Aeromonas hydrophila*	ZnO	Spherical	50–70	Aanti-bacterial against *P. aeruginosa* and *A. flavus*	[115]
*Bacillus mycoides*	TiO_2_	Spherical	40–60	Supress aquatic biofilm growth	[116]
*Proteus mirabilis* PTCC1710	Au	Spherical	10–20	No reported anti-bacterial activity	[117]
*Escherichia coli*	CdS	Spherical	2–5	Anti-bacterial against *E. coli* strain BW25113	[118]
Strains NS2 and NS6	PbS		40–70	Bioremidiation	[119]
**Fungus and Yeast**					
*Volvariella volvacea*	Au and Ag	Spherical/hexagonal	20–150	Anti-bacterial	[120]
*Aspergillus flavus*	TiO_2_	Oval	60–74	Anti-bacterial against *S. aureus*	[121]
MKY3	Ag	Hexagonal	2–5	Anti-bacterial against *S. aureus* and *E. coli*	[122]

## References

[1] Etebu E., Arikekpar I. (2016). Antibiotics: Classification and mechanisms of action with emphasis on molecular perspectives. Int. J. Appl. Microbiol. Biotechnol. Res..

[2] Kohanski M.A., Dwyer D.J., Collins J.J. (2010). How antibiotics kill bacteria: From targets to networks. Nat. Rev. Microbiol..

[3] Padiyara P., Inoue H., Sprenger M. (2018). Global Governance Mechanisms to Address Antimicrobial Resistance. Infect. Dis. Res. Treat..

[4] González-Candelas F., Comas I., Martínez J.L., Galán J.C., Baquero F., Tibayrenc M. (2017). 12–The Evolution of Antibiotic Resistance. Genetics and Evolution of Infectious Diseases.

[5] Pelgrift R.Y., Friedman A.J. (2013). Nanotechnology as a therapeutic tool to combat microbial resistance. Adv. Drug Deliv. Rev..

[6] Ventola C.L. (2015). The antibiotic resistance crisis: Part 1: Causes and threats. P T Peer-Rev. J. Formul. Manag..

[7] Baluja Z., Nabi N., Ray A. (2018). Challenges in Antimicrobial Resistance: An Update. EC Pharmacol. Toxicol..

[8] Davis M., Whittaker A., Lindgren M., Djerf-Pierre M., Manderson L., Flowers P. (2018). Understanding media publics and the antimicrobial resistance crisis. Glob. Public Health.

[9] Betts J.W., Hornsey M., La Ragione R.M. (2018). Novel Antibacterials: Alternatives to Traditional Antibiotics. Adv. Microb. Physiol..

[10] Blecher K., Nasir A., Friedman A. (2011). The growing role of nanotechnology in combating infectious disease. Virulence.

[11] Huh A.J., Kwon Y.J. (2011). “Nanoantibiotics”: A new paradigm for treating infectious diseases using nanomaterials in the antibiotics resistant era. J. Control. Release: Off. J. Controll. Release Soc..

[12] Teixeira M.C., Sanchez-Lopez E., Espina M., Calpena A.C., Silva A.M., Veiga F.J., Garcia M.L., Souto E.B., Shegokar R., Souto E.B. (2018). Chapter 9—Advances in antibiotic nanotherapy: Overcoming antimicrobial resistance. Emerging Nanotechnologies in Immunology.

[13] Bartlett J.G., Gilbert D.N., Spellberg B. (2013). Seven ways to preserve the miracle of antibiotics. Clin. Infect. Dis. Off. Publ. Infect. Dis. Soc. Am..

[14] Adeniji F. (2018). Global analysis of strategies to tackle antimicrobial resistance. Int. J. Pharm. Pract..

[15] Ahn S., Singh P., Jang M., Kim Y.J., Castro-Aceituno V., Simu S.Y., Kim Y.J., Yang D.C. (2018). Gold nanoflowers synthesized using Acanthopanacis cortex extract inhibit inflammatory mediators in LPS-induced RAW264.7 macrophages via NF-kappaB and AP-1 pathways. Colloids Surf. B Biointerfaces.

[16] Singh P., Singh H., Ahn S., Castro-Aceituno V., Jimenez Z., Simu S.Y., Kim Y.J., Yang D.C. (2017). Pharmacological importance, characterization and applications of gold and silver nanoparticles synthesized by Panax ginseng fresh leaves. Artif. Cells Nanomed. Biotechnol..

[17] Singh P., Kim Y.J., Wang C., Mathiyalagan R., Yang D.C. (2016). Microbial synthesis of Flower-shaped gold nanoparticles. Artif. Cells Nanomed. Biotechnol..

[18] Singh P., Kim Y.J., Singh H., Mathiyalagan R., Wang C., Yang D.C. (2015). Biosynthesis of Anisotropic Silver Nanoparticles by *Bhargavaea indica* and Their Synergistic Effect with Antibiotics against Pathogenic Microorganisms. J. Nanomater..

[19] Singh P., Pandit S., Garnaes J., Tunjic S., Mokkapati V.R., Sultan A., Thygesen A., Mackevica A., Mateiu R.V., Daugaard A.E. (2018). Green synthesis of gold and silver nanoparticles from Cannabis sativa (industrial hemp) and their capacity for biofilm inhibition. Int. J. Nanomed..

[20] Singh P., Kim Y.J., Yang D.C. (2016). A strategic approach for rapid synthesis of gold and silver nanoparticles by Panax ginseng leaves. Artif. Cells Nanomed. Biotechnol..

[21] Singh P., Kim Y.J., Wang C., Mathiyalagan R., Yang D.C. (2016). The development of a green approach for the biosynthesis of silver and gold nanoparticles by using Panax ginseng root extract, and their biological applications. Artif. Cells Nanomed. Biotechnol..

[22] Singh P., Kim Y.J., Wang C., Mathiyalagan R., El-Agamy Farh M., Yang D.C. (2016). Biogenic silver and gold nanoparticles synthesized using red ginseng root extract, and their applications. Artif. Cells Nanomed. Biotechnol..

[23] Singh P., Kim Y.J., Zhang D., Yang D.C. (2016). Biological Synthesis of Nanoparticles from Plants and Microorganisms. Trends Biotechnol..

[24] Singh P., Ahn S., Kang J.P., Veronika S., Huo Y., Singh H., Chokkaligam M., El-Agamy Farh M., Aceituno V.C., Kim Y.J. (2017). In vitro anti-inflammatory activity of spherical silver nanoparticles and monodisperse hexagonal gold nanoparticles by fruit extract of *Prunus serrulata*: A green synthetic approach. Artif. Cells Nanomed. Biotechnol..

[25] Abbai R., Mathiyalagan R., Markus J., Kim Y.J., Wang C., Singh P., Ahn S., Farh Mel A., Yang D.C. (2016). Green synthesis of multifunctional silver and gold nanoparticles from the oriental herbal adaptogen: Siberian ginseng. Int. J. Nanomed..

[26] Singh P., Kim Y.J., Wang C., Mathiyalagan R., Yang D.C. (2016). Weissella oryzae DC6-facilitated green synthesis of silver nanoparticles and their antimicrobial potential. Artif. Cells Nanomed. Biotechnol..

[27] Jo J.H., Singh P., Kim Y.J., Wang C., Mathiyalagan R., Jin C.G., Yang D.C. (2016). Pseudomonas deceptionensis DC5-mediated synthesis of extracellular silver nanoparticles. Artif. Cells Nanomed. Biotechnol..

[28] Singh H., Du J., Singh P., Yi T.H. (2018). Ecofriendly synthesis of silver and gold nanoparticles by Euphrasia officinalis leaf extract and its biomedical applications. Artif. Cells Nanomed. Biotechnol..

[29] Huo Y., Singh P., Kim Y.J., Soshnikova V., Kang J., Markus J., Ahn S., Castro-Aceituno V., Mathiyalagan R., Chokkalingam M. (2018). Biological synthesis of gold and silver chloride nanoparticles by Glycyrrhiza uralensis and in vitro applications. Artif. Cells Nanomed. Biotechnol..

[30] Adzitey F. (2015). Antibiotic Classes and Antibiotic Susceptibility of Bacterial Isolates from Selected Poultry; A Mini Review. World’s Vet. J..

[31] Livermore D.M., Warner M., Mushtaq S., Doumith M., Zhang J., Woodford N. (2011). What remains against carbapenem-resistant Enterobacteriaceae? Evaluation of chloramphenicol, ciprofloxacin, colistin, fosfomycin, minocycline, nitrofurantoin, temocillin and tigecycline. Int. J. Antimicrob. Agents.

[32] Blaskovich M.A.T., Hansford K.A., Butler M.S., Jia Z., Mark A.E., Cooper M.A. (2018). Developments in Glycopeptide Antibiotics. ACS Infect. Dis..

[33] Pandit N., Singla R.K., Shrivastava B. (2012). Current Updates on Oxazolidinone and Its Significance. Int. J. Med. Chem..

[34] Dowling A., O’Dwyer J., Adley C. (2017). Antibiotics: Mode of Action and Mechanisms of Resistance.

[35] Cetinkaya Y., Falk P., Mayhall C.G. (2000). Vancomycin-resistant enterococci. Clin. Microbiol. Rev..

[36] Hajipour M.J., Fromm K.M., Akbar Ashkarran A., Jimenez de Aberasturi D., Larramendi I.R.D., Rojo T., Serpooshan V., Parak W.J., Mahmoudi M. (2012). Antibacterial properties of nanoparticles. Trends Biotechnol..

[37] Ganjian H., Nikokar I., Tieshayar A., Mostafaei A., Amirmozafari N., Kiani S. (2012). Effects of Salt Stress on the Antimicrobial Drug Resistance and Protein Pattern of *Staphylococcus aureus*. Jundishapur J. Microbiol..

[38] Jayaraman R. (2009). Antibiotic resistance: An overview of mechanisms and a paradigm shift. Curr. Sci..

[39] Deurenberg R.H., Stobberingh E.E. (2009). The molecular evolution of hospital- and community-associated methicillin-resistant *Staphylococcus aureus*. Curr. Mol. Med..

[40] Périchon B., Courvalin P. (2009). VanA-Type Vancomycin-Resistant *Staphylococcus aureus*. Antimicrob. Agents Chemother..

[41] Deck D.H., Winston L.G., Katzung B., Masters S., Trevor A. (2012). Sulfonamides, trimethoprim, & quinolones. Basic and Clinical Pharmacology.

[42] Poole K. (2002). Mechanisms of bacterial biocide and antibiotic resistance. J. Appl. Microbiol..

[43] Bahar A.A., Ren D. (2013). Antimicrobial peptides. Pharmaceuticals (Basel).

[44] Ferreira C., Pereira A., Melo L., Simões M., Méndez-Vilas A. (2010). Advances in industrial biofilm control with micro-nanotechnology. Current Research, Technology and Education Topics in Applied Microbiology and Microbial Biotechnology.

[45] Munita J.M., Arias C.A. (2016). Mechanisms of Antibiotic Resistance. Microbiol. Spectr..

[46] Fernandez-Moure J.S., Evangelopoulos M., Colvill K., Van Eps J.L., Tasciotti E. (2017). Nanoantibiotics: A new paradigm for the treatment of surgical infection. Nanomed. (Lond.).

[47] Zaidi S., Misba L., Khan A.U. (2017). Nano-therapeutics: A revolution in infection control in post antibiotic era. Nanomed. Nanotechnol. Biol. Med..

[48] Singh P., Kim Y.J., Singh H., Wang C., Hwang K.H., Farh Mel A., Yang D.C. (2015). Biosynthesis, characterization, and antimicrobial applications of silver nanoparticles. Int. J. Nanomed..

[49] Singh P., Singh H., Kim Y.J., Mathiyalagan R., Wang C., Yang D.C. (2016). Extracellular synthesis of silver and gold nanoparticles by *Sporosarcina koreensis* DC4 and their biological applications. Enzyme Microb. Technol..

[50] Sathiyavimal S., Vasantharaj S., Bharathi D., Saravanan M., Manikandan E., Kumar S.S., Pugazhendhi A. (2018). Biogenesis of copper oxide nanoparticles (CuONPs) using *Sida acuta* and their incorporation over cotton fabrics to prevent the pathogenicity of Gram negative and Gram positive bacteria. J. Photochem. Photobiol. B Biol..

[51] Pasquet J., Chevalier Y., Pelletier J., Couval E., Bouvier D., Bolzinger M.-A. (2014). The contribution of zinc ions to the antimicrobial activity of zinc oxide. Colloids Surf. A Physicochem. Eng. Asp..

[52] Mukherjee S., Sushma V., Patra S., Barui A.K., Bhadra M.P., Sreedhar B., Patra C.R. (2012). Green chemistry approach for the synthesis and stabilization of biocompatible gold nanoparticles and their potential applications in cancer therapy. Nanotechnology.

[53] Correa J.M., Mori M., Sanches H.L., da Cruz A.D., Poiate E., Poiate I.A. (2015). Silver nanoparticles in dental biomaterials. Int. J. Biomater..

[54] Burdusel A.C., Gherasim O., Grumezescu A.M., Mogoanta L., Ficai A., Andronescu E. (2018). Biomedical Applications of Silver Nanoparticles: An Up-to-Date Overview. Nanomaterials.

[55] Dakal T.C., Kumar A., Majumdar R.S., Yadav V. (2016). Mechanistic Basis of Antimicrobial Actions of Silver Nanoparticles. Front. Microbiol..

[56] Slavin Y.N., Asnis J., Hafeli U.O., Bach H. (2017). Metal nanoparticles: Understanding the mechanisms behind antibacterial activity. J. Nanobiotechnol..

[57] Vimbela G.V., Ngo S.M., Fraze C., Yang L., Stout D.A. (2017). Antibacterial properties and toxicity from metallic nanomaterials. Int. J. Nanomed..

[58] Hoseinnejad M., Jafari S.M., Katouzian I. (2018). Inorganic and metal nanoparticles and their antimicrobial activity in food packaging applications. Critical Rev. Microbiol..

[59] Aromal S.A., Vidhu V.K., Philip D. (2012). Green synthesis of well-dispersed gold nanoparticles using Macrotyloma uniflorum. Spectrochim. Acta Part A Mol. Biomol. Spectrosc..

[60] Suman T.Y., Rajasree S.R., Ramkumar R., Rajthilak C., Perumal P. (2014). The Green synthesis of gold nanoparticles using an aqueous root extract of *Morinda citrifolia* L.. Spectrochim. Acta Part A Mol. Biomol. Spectrosc..

[61] Sheny D.S., Mathew J., Philip D. (2012). Synthesis characterization and catalytic action of hexagonal gold nanoparticles using essential oils extracted from *Anacardium occidentale*. Spectrochim. Acta Part A Mol. Biomol. Spectrosc..

[62] Abdel-Raouf N., Al-Enazi N.M., Ibraheem I.B.M. (2017). Green biosynthesis of gold nanoparticles using *Galaxaura elongata* and characterization of their antibacterial activity. Arabian J. Chem..

[63] Yang N., WeiHong L., Hao L. (2014). Biosynthesis of Au nanoparticles using agricultural waste mango peel extract and its in vitro cytotoxic effect on two normal cells. Mater. Lett..

[64] Brown A.N., Smith K., Samuels T.A., Lu J., Obare S.O., Scott M.E. (2012). Nanoparticles functionalized with ampicillin destroy multiple-antibiotic-resistant isolates of Pseudomonas aeruginosa and Enterobacter aerogenes and methicillin-resistant Staphylococcus aureus. Appl. Environ. Microbiol..

[65] Suresh A.K., Pelletier D.A., Wang W., Morrell-Falvey J.L., Gu B., Doktycz M.J. (2012). Cytotoxicity induced by engineered silver nanocrystallites is dependent on surface coatings and cell types. Langmuir ACS J. Surf. Colloids.

[66] Kim T., Braun G.B., She Z.G., Hussain S., Ruoslahti E., Sailor M.J. (2016). Composite Porous Silicon-Silver Nanoparticles as Theranostic Antibacterial Agents. ACS Appl. Mater. Interfaces.

[67] Hindi K.M., Ditto A.J., Panzner M.J., Medvetz D.A., Han D.S., Hovis C.E., Hilliard J.K., Taylor J.B., Yun Y.H., Cannon C.L. (2009). The antimicrobial efficacy of sustained release silver-carbene complex-loaded l-tyrosine polyphosphate nanoparticles: Characterization, in vitro and in vivo studies. Biomaterials.

[68] Lara H.H., Ayala-Núñez N.V., Ixtepan Turrent L.D.C., Rodríguez Padilla C. (2010). Bactericidal effect of silver nanoparticles against multidrug-resistant bacteria. World J. Microbiol. Biotechnol..

[69] Raza M.A., Kanwal Z., Rauf A., Sabri A.N., Riaz S., Naseem S. (2016). Size- and Shape-Dependent Antibacterial Studies of Silver Nanoparticles Synthesized by Wet Chemical Routes. Nanomaterials.

[70] Tang S., Zheng J. (2018). Antibacterial Activity of Silver Nanoparticles: Structural Effects. Adv. Healthc. Mater..

[71] Jinu U., Jayalakshmi N., Sujima Anbu A., Mahendran D., Sahi S., Venkatachalam P. (2017). Biofabrication of Cubic Phase Silver Nanoparticles Loaded with Phytochemicals from Solanum nigrum Leaf Extracts for Potential Antibacterial, Antibiofilm and Antioxidant Activities Against MDR Human Pathogens. J. Clust. Sci..

[72] Gopinath P.M., Narchonai G., Dhanasekaran D., Ranjani A., Thajuddin N. (2015). Mycosynthesis, characterization and antibacterial properties of AgNPs against multidrug resistant (MDR) bacterial pathogens of female infertility cases. Asian J. Pharm. Sci..

[73] Shahverdi A.R., Fakhimi A., Shahverdi H.R., Minaian S. (2007). Synthesis and effect of silver nanoparticles on the antibacterial activity of different antibiotics against Staphylococcus aureus and *Escherichia coli*. Nanomed. Nanotechnol. Biol. Med..

[74] Leid J.G., Ditto A.J., Knapp A., Shah P.N., Wright B.D., Blust R., Christensen L., Clemons C.B., Wilber J.P., Young G.W. (2012). In vitro antimicrobial studies of silver carbene complexes: Activity of free and nanoparticle carbene formulations against clinical isolates of pathogenic bacteria. J. Antimicrob. Chemother..

[75] Kaweeteerawat C., Na Ubol P., Sangmuang S., Aueviriyavit S., Maniratanachote R. (2017). Mechanisms of antibiotic resistance in bacteria mediated by silver nanoparticles. J. Toxicol. Environ. Health. A.

[76] Madhumitha G., Elango G., Roopan S.M. (2016). Biotechnological aspects of ZnO nanoparticles: Overview on synthesis and its applications. Appl. Microbiol. Biotechnol..

[77] Ahmed S., Annu, Chaudhry S.A., Ikram S. (2017). A review on biogenic synthesis of ZnO nanoparticles using plant extracts and microbes: A prospect towards green chemistry. J. Photochem. Photobiol. B Biol..

[78] Bhuyan T., Mishra K., Khanuja M., Prasad R., Varma A. (2015). Biosynthesis of zinc oxide nanoparticles from Azadirachta indica for antibacterial and photocatalytic applications. Mater. Sci. Semicond. Process..

[79] Ali K., Dwivedi S., Azam A., Saquib Q., Al-Said M.S., Alkhedhairy A.A., Musarrat J. (2016). Aloe vera extract functionalized zinc oxide nanoparticles as nanoantibiotics against multi-drug resistant clinical bacterial isolates. J. Colloid Interface Sci..

[80] Vimala K., Sundarraj S., Paulpandi M., Vengatesan S., Kannan S. (2014). Green synthesized doxorubicin loaded zinc oxide nanoparticles regulates the Bax and Bcl-2 expression in breast and colon carcinoma. Process. Biochem..

[81] Jones N., Ray B., Ranjit K.T., Manna A.C. (2008). Antibacterial activity of ZnO nanoparticle suspensions on a broad spectrum of microorganisms. FEMS Microbiol. Lett..

[82] Liu Y., He L., Mustapha A., Li H., Hu Z.Q., Lin M. (2009). Antibacterial activities of zinc oxide nanoparticles against Escherichia coli O157:H7. J. Appl. Microbiol..

[83] Tiwari V., Mishra N., Gadani K., Solanki P.S., Shah N.A., Tiwari M. (2018). Mechanism of Anti-bacterial Activity of Zinc Oxide Nanoparticle Against Carbapenem-Resistant Acinetobacter baumannii. Front. Microbiol..

[84] Kumar A., Pandey A.K., Singh S.S., Shanker R., Dhawan A. (2011). Engineered ZnO and TiO(2) nanoparticles induce oxidative stress and DNA damage leading to reduced viability of Escherichia coli. Free Radic. Biol. Med..

[85] Horie M., Fujita K., Kato H., Endoh S., Nishio K., Komaba L.K., Nakamura A., Miyauchi A., Kinugasa S., Hagihara Y. (2012). Association of the physical and chemical properties and the cytotoxicity of metal oxide nanoparticles: Metal ion release, adsorption ability and specific surface area. Metall. Integr. Biomet. Sci..

[86] McDevitt C.A., Ogunniyi A.D., Valkov E., Lawrence M.C., Kobe B., McEwan A.G., Paton J.C. (2011). A molecular mechanism for bacterial susceptibility to zinc. PLoS Pathog..

[87] Li M., Zhu L., Lin D. (2011). Toxicity of ZnO nanoparticles to Escherichia coli: Mechanism and the influence of medium components. Environ. Sci. Technol..

[88] El-Batal A.I., El-Sayyad G.S., El-Ghamery A., Gobara M. (2017). Response Surface Methodology Optimization of Melanin Production by Streptomyces cyaneus and Synthesis of Copper Oxide Nanoparticles Using Gamma Radiation. J. Clust. Sci..

[89] Singh B.P., Jena B.K., Bhattacharjee S., Besra L. (2013). Development of oxidation and corrosion resistance hydrophobic graphene oxide-polymer composite coating on copper. Surf. Coat. Technol..

[90] El-Batal A.I., Al-Hazmi N.E., Mosallam F.M., El-Sayyad G.S. (2018). Biogenic synthesis of copper nanoparticles by natural polysaccharides and Pleurotus ostreatus fermented fenugreek using gamma rays with antioxidant and antimicrobial potential towards some wound pathogens. Microb. Pathog..

[91] Hoshino N., Kimura T., Yamaji A., Ando T. (1999). Damage to the cytoplasmic membrane of *Escherichia coli* by catechin-copper (II) complexes. Free Radic. Biol. Med..

[92] Bogdanović U., Lazić V., Vodnik V., Budimir M., Marković Z., Dimitrijević S. (2014). Copper nanoparticles with high antimicrobial activity. Mater. Lett..

[93] DeAlba-Montero I., Guajardo-Pacheco J., Morales-Sanchez E., Araujo-Martinez R., Loredo-Becerra G.M., Martinez-Castanon G.A., Ruiz F., Compean Jasso M.E. (2017). Antimicrobial Properties of Copper Nanoparticles and Amino Acid Chelated Copper Nanoparticles Produced by Using a Soya Extract. Bioinorg. Chem. Appl..

[94] Borkow G., Gabbay J. (2004). Putting copper into action: Copper-impregnated products with potent biocidal activities. FASEB J. Official Publ. Fed. Am. Soc. Exp. Biol..

[95] Hostynek J.J., Maibach H.I. (2003). Copper hypersensitivity: Dermatologic aspects—An overview. Rev. Environ. Health.

[96] Vamathevan V., Amal R., Beydoun D., Low G., McEvoy S. (2002). Photocatalytic oxidation of organics in water using pure and silver-modified titanium dioxide particles. J. Photochem. Photobiol. A Chem..

[97] Varghese O.K., Gong D., Paulose M., Ong K.G., Grimes C.A. (2003). Hydrogen sensing using titania nanotubes. Sens. Actuators B Chem..

[98] Zhou H., Gan X., Wang J., Zhu X., Li G. (2005). Hemoglobin-based hydrogen peroxide biosensor tuned by the photovoltaic effect of nano titanium dioxide. Anal. Chem.

[99] Zhang J., Li S., Ding H., Li Q., Wang B., Wang X., Wang H. (2014). Transfer and assembly of large area TiO_2_ nanotube arrays onto conductive glass for dye sensitized solar cells. J. Power Sources.

[100] Kirillin M., Shirmanova M., Sirotkina M., Bugrova M., Khlebtsov B., Zagaynova E. (2009). Contrasting properties of gold nanoshells and titanium dioxide nanoparticles for optical coherence tomography imaging of skin: Monte Carlo simulations and in vivo study. J. Biomed. Opt..

[101] Chen X., Mao S.S. (2007). Titanium dioxide nanomaterials: Synthesis, properties, modifications, and applications. Chem. Rev..

[102] Mali S.S., Betty C.A., Bhosale P.N., Patil P.S. (2011). Hydrothermal synthesis of rutile TiO_2_ with hierarchical microspheres and their characterization. CrystEngComm.

[103] Sankar R., Rizwana K., Shivashangari K.S., Ravikumar V. (2015). Ultra-rapid photocatalytic activity of *Azadirachta indica* engineered colloidal titanium dioxide nanoparticles. Appl. Nanosci..

[104] Rajakumar G., Rahuman A.A., Roopan S.M., Khanna V.G., Elango G., Kamaraj C., Zahir A.A., Velayutham K. (2012). Fungus-mediated biosynthesis and characterization of TiO_2_ nanoparticles and their activity against pathogenic bacteria. Spectrochim. Acta A Mol. Biomol. Spectrosc..

[105] Hunagund S.M., Desai V.R., Kadadevarmath J.S., Barretto D.A., Vootla S., Sidarai A.H. (2016). Biogenic and chemogenic synthesis of TiO_2_ NPs via hydrothermal route and their antibacterial activities. RSC Adv..

[106] Foster H.A., Ditta I.B., Varghese S., Steele A. (2011). Photocatalytic disinfection using titanium dioxide: Spectrum and mechanism of antimicrobial activity. Appl. Microbiol. Biotechnol..

[107] Li Y., Zhang W., Niu J., Chen Y. (2012). Mechanism of photogenerated reactive oxygen species and correlation with the antibacterial properties of engineered metal-oxide nanoparticles. ACS Nano.

[108] Leung Y.H., Ng A.M., Xu X., Shen Z., Gethings L.A., Wong M.T., Chan C.M., Guo M.Y., Ng Y.H., Djurisic A.B. (2014). Mechanisms of antibacterial activity of MgO: Non-ROS mediated toxicity of MgO nanoparticles towards *Escherichia coli*. Small.

[109] Singh P., Pandit S., Mokkapati V., Garg A., Ravikumar V., Mijakovic I. (2018). Gold Nanoparticles in Diagnostics and Therapeutics for Human Cancer. Int. J. Mol. Sci..

[110] Singh K., Panghal M., Kadyan S., Chaudhary U., Yadav J.P. (2014). Green silver nanoparticles of *Phyllanthus amarus*: As an antibacterial agent against multi drug resistant clinical isolates of *Pseudomonas aeruginosa*. J. NanoBiotechnol..

[111] Ordenes-Aenishanslins N.A., Saona L.A., Duran-Toro V.M., Monras J.P., Bravo D.M., Perez-Donoso J.M. (2014). Use of titanium dioxide nanoparticles biosynthesized by Bacillus mycoides in quantum dot sensitized solar cells. Microb. Cell Fact..

[112] Korbekandi H., Iravani S., Abbasi S. (2009). Production of nanoparticles using organisms. Crit. Rev. Biotechnol..

[113] Velayutham K., Rahuman A.A., Rajakumar G., Santhoshkumar T., Marimuthu S., Jayaseelan C., Bagavan A., Kirthi A.V., Kamaraj C., Zahir A.A. (2012). Evaluation of Catharanthus roseus leaf extract-mediated biosynthesis of titanium dioxide nanoparticles against *Hippobosca maculata* and *Bovicola ovis*. Parasitol. Res..

[114] Choi O., Deng K.K., Kim N.J., Ross L., Surampalli R.Y., Hu Z. (2008). The inhibitory effects of silver nanoparticles, silver ions, and silver chloride colloids on microbial growth. Water Res..

[115] Chaloupka K., Malam Y., Seifalian A.M. (2010). Nanosilver as a new generation of nanoproduct in biomedical applications. Trends Biotechnol..

[116] Liu H.L., Dai S.A., Fu K.Y., Hsu S.H. (2010). Antibacterial properties of silver nanoparticles in three different sizes and their nanocomposites with a new waterborne polyurethane. Int. J. Nanomed..

[117] Samadi N., Golkaran D., Eslamifar A., Jamalifar H., Fazeli M.R., Mohseni F.A. (2009). Intra/extracellular biosynthesis of silver nanoparticles by an autochthonous strain of Proteus mirabilis isolated from photographic waste. J. Biomed. Nanotechnol..

[118] Mirzajani F., Ghassempour A., Aliahmadi A., Esmaeili M.A. (2011). Antibacterial effect of silver nanoparticles on Staphylococcus aureus. Res. Microbiol..

[119] Lok C.N., Ho C.M., Chen R., He Q.Y., Yu W.Y., Sun H., Tam P.K., Chiu J.F., Che C.M. (2006). Proteomic analysis of the mode of antibacterial action of silver nanoparticles. J. Proteome Res..

[120] Jung W.K., Koo H.C., Kim K.W., Shin S., Kim S.H., Park Y.H. (2008). Antibacterial activity and mechanism of action of the silver ion in Staphylococcus aureus and *Escherichia coli*. Appl. Environ. Microbiol..

[121] Schreurs W.J., Rosenberg H. (1982). Effect of silver ions on transport and retention of phosphate by *Escherichia coli*. J. Bacteriol..

[122] Apte M., Sambre D., Gaikawad S., Joshi S., Bankar A., Kumar A.R., Zinjarde S. (2013). Psychrotrophic yeast *Yarrowia lipolytica* NCYC 789 mediates the synthesis of antimicrobial silver nanoparticles via cell-associated melanin. AMB Express.

